# Can psychological flexibility and prosociality mitigate illness perceptions toward COVID-19 on mental health? A cross-sectional study among Hong Kong adults

**DOI:** 10.1186/s12992-021-00692-6

**Published:** 2021-04-08

**Authors:** Yuen Yu Chong, Wai Tong Chien, Ho Yu Cheng, Angelos P. Kassianos, Andrew T. Gloster, Maria Karekla

**Affiliations:** 1 The Nethersole School of Nursing, Faculty of Medicine, The Chinese University of Hong Kong, Shatin, Hong Kong; 2 Department of Applied Health Research, University College London, London, UK; 3 Department of Psychology, University of Cyprus, Nicosia, Cyprus; 4 Division of Clinical Psychology and Intervention Science, University of Basel, Basel, Switzerland

**Keywords:** Psychological flexibility, Prosociality, Mental health, Coronavirus, COVID-19

## Abstract

**Background:**

The negative impact of COVID-19 pandemic on public mental health can be persistent and substantial over a long period of time, but little is known regarding what psychological factors or processes can buffer such impact. The present study aimed to examine the mediating roles of coping, psychological flexibility and prosociality in the impacts of perceived illness threats toward COVID-19 on mental health.

**Method:**

Five-hundred and fourteen Hong Kong citizens (18 years or above) completed an online survey to measure illness perceptions toward COVID-19, coping, psychological flexibility, prosociality, and mental health, together with their socio-demographic variables. Structural equation modelling was used to explore the explanatory model that was the best-fit to illustrate the relationships between these constructs.

**Results:**

Serial mediation structural equation model showed that only psychological flexibility (unstandardised beta coefficient, β = − 0.12, 95% CI [− 0.20, − 0.02], *p* = 0.031) and prosociality (unstandardised β = 0.04, 95% CI [0.01, 0.08], *p* = 0.001) fully mediated the relationship between illness perceptions toward COVID-19 and mental health. In addition, psychological flexibility exerted a direct effect on prosociality (standardised β = 0.22, 95% CI [0.12, 0.32], *p* < 0.001). This best-fit model explained 62% of the variance of mental health.

**Conclusions:**

Fostering psychological flexibility and prosocial behaviour may play significant roles in mitigating the adverse effects of COVID-19 and its perceived threats on public mental health.

## Introduction

Ever since the declaration of the coronavirus 2019 (COVID-19) outbreak as a public health emergency, with more than 61.6 million confirmed cases and 1.4 million deaths as of November 28, 2020, there have been reports regarding the detrimental impacts of the pandemic on public mental health [1–3]. Evidence from systematic reviews has indicated that over one-third of the global populations report high levels of psychological distress [2, 3]. Since the first confirmed case of COVID-19 in Hong Kong on 23 January 2020, varied levels of anxiety and stress have been reported in the community [4–6]. The increased incidence of post-traumatic stress disorder and suicide among people in Hong Kong during the 2003 severe acute respiratory syndrome (SARS) epidemic provides clear and affirmed examples of the adverse consequences on public mental health, which may likely be mirrored or intensified during the COVID-19 pandemic [7]. The number of COVID-19 cases in Hong Kong has been exceeding 6000 which surpassed the 2003’s SARS number as of November 28, 2020 [8]. It is expected that the adverse mental health impacts of COVID-19 on the public would likely be widely affected and sustained over a longer period of time beyond the peaks of the pandemic.

Illness perceptions are defined as cognitive and emotional representations or beliefs that an individual has about an illness, which is developed through the information that the individual receives from formal and informal resources [9]. It is well known that such perceptions play important roles in influencing health behaviours and mental health outcomes [10–13]. As stated in the Leventhal’s Common Sense Model of Self-Regulation [14], an infection outbreak can activate an individual’s schema or perception of the illness. A dynamic self-regulatory process of the individual will then be initiated, attempting to use either or both adaptive and maladaptive coping responses to manage the perceived threats concerning the illness and their concomitant emotional reactions arising. The success of this coping process would affect the individual’s health outcomes [14]. Hence, putting into the context of the COVID-19 pandemic, it is plausible that how COVID-19 as perceived by the public could significantly affect their coping responses and health outcomes.

### Psychological flexibility and prosociality

Recent studies have been put forth to identify various sociodemographic factors, social and job-related factors (e.g., working as health care professions, poor household income and high social media exposure) and pre-existing psychiatric illnesses, which increased the risks of anxiety and depression during the COVID-19 pandemic [1, 3, 15]. In addition, there is a growing body of evidence addressing the interrelationships between the perceived threats related to COVID-19, coping strategies and mental health outcomes [16–19]. With the more use of emotional-focused coping [18, 19], the less use of problem-focused coping [19] and the lower social support [18], people tend to exhibit more mental health symptoms, including symptoms of depression and anxiety [18, 19]. However, little is known regarding other potentially modifiable psychosocial factors, apart from various types of coping strategies, which may help to mitigate the mental health impact of COVID-19. Psychological flexibility, which refers to the capacity to be open to difficult experiences while engaging in behaviours consistent with self-chosen values [20], is one of the aforementioned constructs which has been consistently associated with better mental health outcomes under different population groups and contexts [20–22]. Recent studies have also shown that psychological flexibility can alleviate the adversities or negative impacts of recent life stressors on mental health and well-being [23, 24]. When psychological flexibility and coping are concurrently examined, psychological flexibility has been shown to account for a greater proportion of psychological distress over and above an individual’s coping style alone [25–27]. This implies the need to reappraise whether psychological flexibility is an overarching psychological process on top of other adaptive/maladaptive coping processes in helping people to respond effectively to situational demands arising from the pandemic.

Prosociality is defined as a set of attitudes and/or voluntary actions, positive and friendly behaviours that an individual may adopt to help, take care of and comfort others [28]. Its role has been recently discussed in the context of epidemic containment [15, 29–32]. Recent studies have suggested that vaccinating can be interpreted and promoted as a prosocial act, in which adding prosocial messaging into influenza vaccination intervention may drive people to get vaccinated not only because of self-interest, but also for the benefits towards their families, neighbourhood and communities [29, 30]. In the COVID-19 pandemic, prosociality has been advocated as an important therapeutic target, because it has been positively linked with social connections, cohesiveness [33] and better adherence to COVID-19 precautionary measures, because people consider their actions can bring societal and communal benefits (e.g., wearing a face mask to prevent COVID-19 spread) rather than benefiting the self only [15, 31, 32]. In addition, prosociality expressed as engaging in affiliating behaviours or nurturing others has been considered as an effective way of coping when experienced distress through influencing neuro-physiological systems, such as the oxytocin and reward circuitry system in the brain [34, 35]. Therefore, prosociality was hypothesised to be an effective coping strategy to the perceived threats of COVID-19 and might play a role in reducing the related psychological distress.

### Hypothetical model

**Fig. 1:**
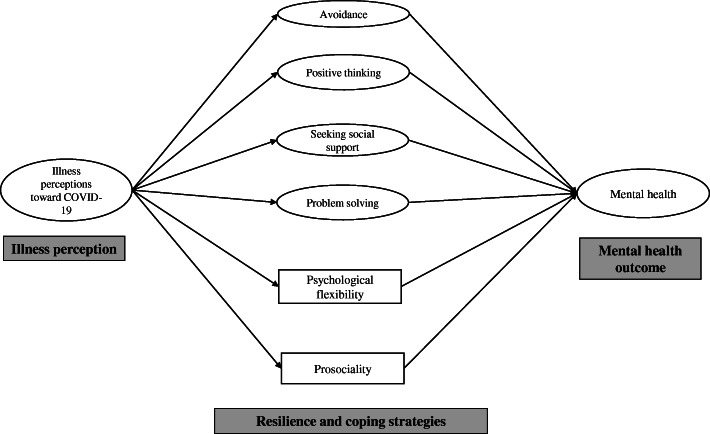
A hypothesised model

In the present study, we followed the hypotheses based on the Leventhal’s Common Sense Model of Self-Regulation, which posits that perceptions of an illness threat (i.e., COVID-19) will motivate various coping strategies, including avoidance, positive thinking, seeking social support and problem solving, to mitigate the threat and affect mental health of an individual [14]. Furthermore, we additionally included psychological flexibility and prosociality to see whether they demonstrate mediating roles in the model in the presence of the above coping factors (see Fig. 1). Identifying the specific pathways of these relations is important not only to acquire a further understanding on how the public psychologically responds to the COVID-19 pandemic but also, more importantly, inform the development of mechanism- or model-based psychological interventions adopting specific coping factors accounting for the plausible adverse effects of COVID-19 and its perceived threats on mental health [33].

## Methods

### Participants and procedures

The present cross-sectional survey was part of the international COVID-IMPACT study (see https://ucy.ac.cy/acthealthy/en/covid-19-impact-survey) and the methodological description of the study conducted in one of 78 study sites (Hong Kong) has been previously reported [36]. Briefly, between April and June 2020, a total of 514 Chinese-speaking Hong Kong residents aged 18 years were conveniently recruited through the highly accessed and commonly used social media platforms (e.g., Facebook, Instagram, Twitter, and WeChat), and the participating universities’ mass emailing. Participants who self-selected and enrolled into the cross-sectional study completed a 20-min online survey via a secured Google platform in which an informed consent was secured on the first page. The ethics approvals of the study were obtained from the Cyprus National Bioethics Committee (ΕΕΒΚ ΕΠ 2020.01.60) and the University in Hong Kong (Reference Number: SBRE-19-593).

### Measures

#### Mental health

The Mental Health Continuum Short Form for Adults (MHC-SF, 14 items, 6-point Likert scale) was used to assess the mental health of the participants, focusing on emotional, social and psychological well-being [37]. For each subscale, higher score indicates better mental health. The MHC-SF subscales possessed good convergent validity and internal consistencies (Cronbach’s α = 0.83–0.89) in both Chinese and Western samples [37, 38] and our sample (α = 0.81–0.88).

#### Illness perceptions toward COVID-19

The Brief Illness Perception Questionnaire (IPQ) items assessing the perceived consequences, timeline, concern and emotional responses toward COVID-19 (4 items, 10-point Likert scale) [9], as well as the items assessing the perceived susceptibility (3 items, 6-point Likert scale) and severity of COVID-19 (3 items, 6-point Likert scale) according to the Health Belief Model [39] were used to assess the public perceptions toward COVID-19 [36]. A higher score indicates a stronger illness perception. The Chinese version of the aforementioned question items showed adequate psychometric properties as stated in our previous report [36].

#### Coping

The Brief Coping Orientation to Problems Experienced (Brief COPE) inventory was adopted to assess the strategies used by the individuals to cope with problems and stress [40]. The instrument includes a total of 28 items, each item could be scored from one (“I haven’t been doing this at all”) to four (“I’ve been doing this a lot”) referring to the following 14 indicators of coping: venting, use of emotional support, use of instrumental support, religion, active coping, planning, behavioural disengagement, self-distraction, substance use, use of denial, self-blame, humour, positive reframing and acceptance. These coping strategies have been recently consolidated and validated as four coping dimensions which are avoidance (5 indicators), positive thinking (3 indicators), seeking social support (4 indicators) and problem solving (2 indicators) [41–43]. The Brief COPE showed adequate convergent validity [40] and has been used in Hong Kong samples [36, 40]. The indicators representing each corresponding coping dimension were demonstrated to have acceptable internal consistencies (α = 0.73–0.82) in our study sample.

#### Psychological flexibility

The PsyFlex questionnaire was used to assess all the six processes of psychological flexibility, including contacting the present moment, defusion, acceptance, self-as-context, values and committed action, of an individual (6 items, 5-point Likert scale) [44, 45]. Example item is “*Even if I am somewhere else with my thoughts, in important moments I can focus on what’s going on at that time*”. Each item could be score from one (“Very often”) to five (“Very seldom). The total score was reversed so that higher score was indicative of greater psychological flexibility. The PsyFlex question items showed good internal consistency in our study sample (α = 0.83).

#### Prosociality

Six items (5-point Likert scale) adapted from the Prosocialness Scale were used to assess prosociality in terms of prosocial behaviours, including sharing, helping, taking care of, and feeling empathic with others, which were carried out by the participants during the COVID-19 pandemic [46]. Example item is “*I am pleased to help my friends/colleagues in their activities”.* Higher total score indicates better prosociality. The items demonstrated acceptable internal consistency (α = 0.83) in our study sample.

The participants were also asked to report their sociodemographic characteristics, impacts of social isolation measures on daily activities (example item: “*Since the social isolation measures began, how frequent you needed to leave your house?*”) and financial situations (example item: “*Since the social isolation measures began, have your financial situation changed?*”), and whether they and/or their family members were infected by COVID-19.

### Statistical analyses

**Fig. 2:**
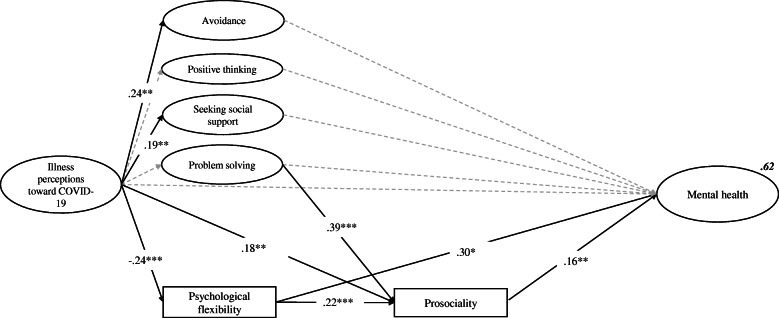
The final structural equation model. *Note.* Latent variables are represented by ellipses. Solid lines indicate significant paths of direct effects, dashed lines indicate non-significant paths of direct effects. For simplicity, the observed variables, covariance paths for all latent variables and significant confounders adjusted for the analysis were not displayed. Standardised path coefficients are presented. *P < .05, ** *P* < .01, *** *P* < .001

Before testing the proposed model, the data were screened for univariate normality and multivariate outliers were detected by the Mahalanobis distance at *p* = .001. Inter-correlations among all the observed variables for the constructs as shown in Fig. 1 were assessed. As suggested by Anderson and Gerbing [47], confirmatory factor analyses (CFA) were first performed to establish the measurement models of the latent constructs, including illness perceptions toward COVID-19, coping (i.e., avoidance, positive thinking, seeking social support, problem solving) and mental health. A structural equation model (SEM) was then built based on the hypothesised model (see Fig. 1) and further analysed with the adjustment for potential confounders, including age, gender, educational level, employment status, having children (yes/no), living conditions, working as health care professionals (yes/no), COVID-19 status (yes/no/unknown) and family members’ COVID-19 status (yes/no/unknown). The model was trimmed by dropping insignificant confounders subsequently. Attempts were made to improve the goodness of fit of the model by adding covariance paths and/or direct paths to explore the interrelationships between the aforementioned latent constructs, psychological flexibility and prosociality, if a significant modification index (MI) coincided with a large expected parameter change (EPC) value [48] (see Fig. 2 for the final model). Biased-corrected and accelerated bootstrapping method with 5000 replications was used to estimate 95% confidence intervals for standardised direct, indirect and total effects. The SEM analyses were estimated by the maximum likelihood method, with the model fit indices [Comparative Fit Index (CFI) ≥ 0.90; Tucker-Lewis Index (TLI) ≥ 0.90; standardised root means square residual (SRMR) ≤ 0.10; and root mean square error approximation (RMSEA) ≤ 0.08] indicating an acceptable model fit [49]. The SPSS AMOS version 23.0 (IBM Corp., Chicago) was used for performed CFA and SEM, while all other statistical analyses were conducted using IBM SPSS version 25.0 (IBM Crop., Armonk, NY). All statistical tests were two-sided and a *p*-value < 0.05 was considered statistically significant.

## Results

The characteristics of the participants have been described and tabulated in our previous report [36]. In brief, the mean (SD) age of the participants was 32.8 (11.5), and 74.1% were females. The majority were graduated from university (81.9%), working as non-health care professionals (74.1%) and living with their parents or own families (79.7%). Approximately one-third of the participants reported that they had been self-isolated (33.7%) and their finance situation became worse since the imposed isolation measures (30.0%). Only one participant reported that he/she was infected by COVID-19, while a small number of the participants (*n* < 5) indicated that they were not sure whether they or their family members were infected by COVID-19 despite of the presence of associated symptoms.

Table 1 presents the descriptive statistics and correlation matrix of the study variables. Significant correlations with medium effect sizes in terms of absolute *r* > .30 found when all indicators assessing mental health (i.e., emotional, social and psychological well-being) correlated with psychological flexibility (*r*s = .49–.60, all *p*s < 0.01) and positive reframing (*r*s = .30–.41, all *p*s < 0.01), respectively. In addition, psychological flexibility significantly correlated with all indicators assessing problem solving (*r*s = .34–.37, all *p*s < 0.01), behavioural disengagement (*r* = −.30, *p* < 0.01), acceptance (*r* = .36, *p* < 0.01) and prosociality (*r* = .35, *p* < 0.01). The results of the CFA indicated that the measuring items corresponding to the latent constructs were all adequately fit (see Table 2).

**Table 1:** Means, standard deviations, ranges and correlations for all study variables

		Variable, correlation																								
Variable (No.)	Mean (SD) [range]	1	2	3	4	5	6	7	8	9	10	11	12	13	14	15	16	17	18	19	20	21	22	23	24	25
Mental health																										
Emotional well-being (1)	8.73 (3.06) [0–15]	1																								
Social well-being (2)	8.35 (5.05) [0–25]	.517^**^	1																							
Psychological well-being (3)	17.15 (6.46) [0–30]	.662^**^	.591^**^	1																						
Illness perceptions toward COVID-19																										
HBM - Perceived severity (4)	14.55 (3.02) [3–18]	−.055	−.189^**^	−0.024	1																					
HBM - Perceived susceptibility (5)	9.23 (2.99) [3–17]	−.134^**^	−.147^**^	−.122^**^	.404^**^	1																				
IPQ – Concern (6)	6.63 (2.03) [1–10]	−.136^**^	−.156^**^	−.087^*^	.485^**^	.391^**^	1																			
IPQ – Consequences (7)	6.84 (1.88) [1–10]	−.139^**^	−.140^**^	−.124^**^	.225^**^	.218^**^	.413^**^	1																		
IPQ - Emotional response (8)	6.28 (2.09) [1–10]	−.078	−.037	−.040	.166^**^	.741^**^	.200^**^	.147^**^	1																	
IPQ – Timeline (9)	7.09 (1.64) [1–10]	−.051	−.148^**^	−.040	.145^**^	.192^**^	.274^**^	.267^**^	.164^**^	1																
Seeking social support																										
Use of emotional support (10)	4.75 (1.59) [2–8]	.024	.079	.113^*^	.119^**^	.107^*^	.093^*^	.094^*^	−.011	.025	1															
Use of instrumental support (11)	5.28 (1.52) [2–8]	.109^*^	.151^**^	.190^**^	.122^**^	.074	.084	.071	−.002	−.043	.768^**^	1														
Venting (12)	5.23 (1.45) [2–8]	.013	.027	.146^**^	.110^*^	.083	.049	.088^*^	.025	−.045	.566^**^	.570^**^	1													
Religion (13)	4.06 (1.92) [2–8]	.130^**^	.221^**^	.156^**^	−.034	.037	−.071	−.049	.042	.010	.230^**^	.253^**^	.122^**^	1												
Problem solving																										
Active coping (14)	5.78 (1.44) [2–8]	.256^**^	.264^**^	.383^**^	.061	−.055	−.053	−.055	−.012	−.070	.333^**^	.376^**^	.320^**^	.191^**^	1											
Planning (15)	5.90 (10.34) [2–8]	.185^**^	.199^**^	.351^**^	.124^**^	.003	−.060	−.008	.020	−.023	.341^**^	.399^**^	.351^**^	.206^**^	.641^**^	1										
Avoidance																										
Self-distraction (16)	5.19 (1.54) [2–8]	−.016	.026	.087^*^	.115^**^	.054	.149^**^	.141^**^	.003	.006	.417^**^	.407^**^	.362^**^	.098^*^	.388^**^	.282^**^	1									
Denial (17)	2.84 (1.16) [2–7]	−.112^*^	.046	−.136^**^	−.026	.094^*^	.146^**^	.090^*^	.093^*^	.023	.115^**^	.097^*^	.089^*^	.021	−.029	−.035	.163^**^	1								
Substance use (18)	2.69 (1.34) [2–8]	−.077	−.001	−.026	−.019	.097^*^	.054	.150^**^	.105^*^	.092^*^	.115^**^	.136^**^	.026	.061	.038	.052	.066	.115^**^	1							
Behavioural disengagement (19)	3.40 (1.26) [2–8]	−.227^**^	−.087^*^	−.263^**^	−.073	.074	−.015	.045	.023	.009	.237^**^	.152^**^	.159^**^	.032	−.095^*^	−.039	.106^*^	.391^**^	.119^**^	1						
Self-blaming (20)	4.33 (1.49) [2–8]	−.253^**^	−.117^**^	−.215^**^	.086	.098^*^	.093^*^	.019	−.003	−.056	.355^**^	.291^**^	.320^**^	.124^**^	.117^**^	.212^**^	.240^**^	.240^**^	.089^*^	.483^**^	1					
Positive thinking																										
Humour (21)	4.04 (1.54) [2–8]	.075	.005	.098^*^	.021	.018	−.084	.032	.057	.062	.163^**^	.123^**^	.269^**^	.101^*^	0.073	.206^**^	.113^*^	.108^*^	0.058	.206^**^	.211^**^	1				
Positive reframing (22)	5.50 (1.45) [2–8]	.298^**^	.306^**^	.405^**^	−.024	−.019	−.069	−.056	.049	−.100^*^	.216^**^	.320^**^	.297^**^	.267^**^	.554^**^	.540^**^	.291^**^	.013	−.010	−.061	.047	.163^**^	1			
Acceptance (23)	6.17 (1.34) [2–8]	.260^**^	.117^**^	.374^**^	.142^**^	.035	−.084	−.065	.046	−0.019	.150^**^	.271^**^	.326^**^	.114^**^	.469^**^	.552^**^	.219^**^	−.162^**^	−.030	−.136^**^	.021	.211^**^	.484^**^	1		
Psychological flexibility																										
Total score (24)	19.40 (4.02) [6–30]	.489^**^	.407^**^	.602^**^	−.084	−.181^**^	−.153^**^	−.111^*^	−.054	−.101^*^	−.002	.088^*^	.025	.152^**^	.369^**^	.336^**^	.077	−.102^*^	−.092^*^	−.301^**^	−.263^**^	.043	.394^**^	.363^**^	1	
Prosociality																										
Total score (25)	20.72 (3.92) [6–30]	.222^**^	.270^**^	.379^**^	.060	−.083	.030	.093^*^	−.069	.003	.345^**^	.345^**^	.271^**^	.144^**^	.341^**^	.389^**^	.222^**^	−.019	−.011	−.050	.114^**^	.107^*^	.306^**^	.301^**^	.347^**^	1

*COVID-19* coronavirus disease 2019, *HBM* Health Belief Model, *IPQ* Illness Perception Questionnaire. For each variable, a high score indicates a greater extent of the underlying trait being measured

^*^ *P* < .05, calculated using 2-tailed bivariate correlations

^**^ *P* < .01, calculated using 2-tailed bivariate correlations

**Table 2:** Measurement models of the latent constructs included in the structural equation model

Latent constructs / indicators	CFA goodness-of-fit indices
Illness perceptions toward COVID-19	χ^2^ = 13.98, RMSEA = 0.06, CFI = 0.91, SRMR = 0.06
HBM - Perceived severity	
HBM - Perceived susceptibility	
IPQ – Consequences	
IPQ – Timeline	
IPQ - Concern	
IPQ – Emotional response	
Avoidance	χ^2^ = 10.4, RMSEA = 0.06, CFI = 0.98, SRMR = 0.04
Self-distraction	
Denial	
Substance use	
Behavioural disengagement	
Self-blaming	
Positive thinking	χ^2^ = 3.8, RMSEA = 0.07, CFI = 0.98, SRMR = 0.06
Humour	
Positive reframing	
Acceptance	
Seeking social support	χ^2^ = 3.2, RMSEA = 0.03, CFI = 1.00, SRMR = 0.02
Use of emotional support	
Use of instrumental support	
Venting	
Religion	
Problem solving	χ^2^ = 4.9, RMSEA = 0.04, CFI = 0.91, SRMR = 0.06
Active coping	
Planning	
Mental health	χ^2^ = 4.9, RMSEA = 0.04, CFI = 1.00, SRMR = 0.06
Emotional well-being	
Social well-being	
Psychological well-being	

*CFI* Comparative Fit Index, *COVID-19* coronavirus disease 2019, *HBM* Health Belief Model*, IPQ* Illness Perception Questionnaire, *RMSEA* root mean square error approximation, *SRMR* standardized root means square residual

Table 3 shows the progression of model modifications and model fit indices. In view of the fairly fitted model (see Model 1 in Table 3) and the purpose of exploratory analyses in identifying the interrelationships between coping, psychological flexibility and prosociality, we followed the suggestions based on the modification indices and expected parameter changes by adding in two direct paths, which were (1) from problem solving to prosociality (MI = 108.74, EPC = 1.74) and (2) from psychological flexibility to prosociality (MI = 16.63, EPC = 0.52), to improve the model fit. After the inclusion of the direct paths, the final SEM (see Model 3 in Table 3) was tested and demonstrated a reasonable good fit with the data (χ^2^ = 878.0, *df* = 294, CFI = 0.92, TLI = 0.90, SRMR = 0.07, RMSEA = 0.06).

**Table 3:** Progression of the model modifications and model fit indices

Model	Model details and modifications	χ^2^ (*df*)	CFI	TLI	SRMR	RMSEA (90% CI)
1	6 latent constructs (illness perceptions toward COVID-19, avoidance, positive thinking, seeking social support, problem solving and mental health), 2 observed variables (psychological flexibility, prosociality) and 2 significant confounders (gender, working as health care professionals) remained after trimming by removing insignificant confounders	960.23 (296)	0.83	0.83	0.09	0.07 (0.06, 0.08)
2	Direct path from problem solving to prosociality	899.7 (295)	0.87	0.86	0.07	0.06 (0.05, 0.07)
3	Direct path from psychological flexibility to prosociality	878.0 (294)	0.92	0.90	0.07	0.06 (0.06, 0.07)

*CFI* Comparative Fit Index, *COVID-19* coronavirus disease 2019, *RMSEA* root mean square error approximation, *SRMR* standardized root means square residual, *TLI* Tucker Lewis Index

Figure 2 illustrates the final SEM adjusted for significant confounders, including gender and working as health care professionals. Illness perceptions toward COVID-19 was significantly associated with avoidance (standardised beta coefficient, β = 0.24, 95% CI [0.09, 0.42], *p* = 0.002), seeking social support (β = 0.19, 95% CI [0.08, 0.30], *p* = 0.002), prosociality (β = 0.18, 95% CI [0.09, 0.28], *p* = 0.001) and psychological flexibility (β = − 0.24, 95% CI [− 0.33, − 0.15], *p* < 0.001). Also, psychological flexibility (β = 0.30, 95% CI [0.05, 0.51], *p* = 0.04) and prosociality (β = 0.16, 95% CI [0.06, 0.24], *p* = 0.001) were both significantly associated with mental health, while the rest of the coping factors remained non-significant (all *p*s = 0.072–0.415). In addition, psychological flexibility was significantly associated with prosociality (β = 0.22, 95% CI [0.12, 0.32], *p* < 0.001). Table 4 summarises the direct, indirect and total effects of illness perceptions toward COVID-19 on mental health based on the final trimmed SEM. This model showed that only psychological flexibility (unstandardised beta coefficient, β = − 0.12, 95% CI [− 0.20, − 0.02], *p* = 0.031) and prosociality (unstandardised β = 0.04, 95% CI [0.01, 0.08], *p* = 0.001) fully mediated the relationship between illness perceptions toward COVID-19 and mental health, other coping factors did not demonstrate their mediating effects (all *p*s = 0.053–0.381). In overall, the model explained 62% of the variance of mental health.

**Table 4:** Direct, indirect and total effects of illness perceptions toward COVID-19 on mental health

Paths	Unstandardised path coefficient, β Estimate (95% CI)	Standardised path coefficient, β Estimate (95% CI)	*P* value
Direct effect			
IP ➔ Mental health	−0.12 (0.50, 0.11)	−0.10 (−0.39, 0.08)	0.173
Indirect effects			
IP ➔ Avoidance ➔ Mental health	0.06 (−0.01, 0.17)	NA	0.053
IP ➔ Positive thinking ➔ Mental health	0.03 (−0.06, 0.86)	NA	0.381
IP ➔ Seeking social support ➔ Mental health	−0.09 (− 0.41, 0.09)	NA	0.124
IP ➔ Problem solving ➔ Mental health	−0.08 (−1.02, 0.05)	NA	0.194
IP ➔ Psychological flexibility ➔ Mental health	−0.12 (− 0.20, − 0.02)	NA	0.031
IP ➔ Prosociality ➔ Mental health	0.04 (0.01, 0.08)	NA	0.001
IP ➔ Psychological flexibility ➔ Prosociality ➔ Mental health	−0.02 (− 0.03, − 0.01)	NA	0.014
Total effect			
IP ➔ Mental health	−0.28 (− 0.45, − 0.12)	−0.21 (− 0.33, − 0.10)	< 0.001

*COVID-19* coronavirus disease 2019, *CI* confidence interval, *IP* illness perceptions towards COVID-19, *NA* not applicable

## Discussion

This study sought to examine the roles of coping styles, psychological flexibility and prosociality in mitigating the impact of illness perceptions toward COVID-19 on mental health among a sample of Hong Kong adults. Our analysis showed the significant but weak correlations between illness perceptions toward COVID-19 and mental health. Nevertheless, prosociality and psychological flexibility showed the full mediation effects on the relationship between the two aforementioned constructs. These findings are congruent with the theoretical underpinning of the Leventhal’s Common Sense Model of Self-Regulation [14], showing that the psychological impact of health-related stressful events, such as the outbreaks of infectious diseases, is influenced not solely by the specific beliefs about the illness, but also based on the selection of various coping responses to manage the threat in order to restore emotional equilibrium and maintain well-being. More importantly, we found that psychological flexibility and prosociality accounted for significant mediating roles over and above the contributions of other known coping-style variables as found in the framework (i.e., avoidance, positive thinking, seeking social support and problem solving). As shown in our study, the role of psychological flexibility as a protective psychological resource is consistent with the growing body of evidence indicating positive relationship between psychological flexibility and mental health among individuals when experiencing major life stressors [23] and facing outbreaks of infectious diseases like COVID-19 [50–52]. By definition, psychological flexibility refers to an ability to respond effectively to situational demands for pursuing longer-term goal driven by values [20], which requires an individual to have an openness to difficult psychological experiences and an awareness of engaging behavioural changes that are necessary to achieve a valued outcome [20]. Hence, psychological flexibility has been conceptualised as a *higher-order* response style, which may facilitate the selection of adaptive coping responses and related behaviours when facing challenges. This echoes with recent mediational analyses by Dawson and Golijani-Moghaddam (2020) highlighting the role of psychological flexibility in mental health among the people in the United Kingdom [50], and our findings further support that psychological flexibility is independent of, but overlapping with other coping responses [25, 53]. In addition, in view of the unfamiliar contexts of COVID-19 outbreaks, people may not be able to successfully draw their usual repertoire of coping that attenuate stress in a short-term. This may be one of the plausible reasons explaining why other coping factors in our model did not show the mediating roles between people’s illness perceptions toward COVID-19 and their mental health.

Notably, the findings of the present study highlight a significant role of prosociality in accounting for the impact of psychological flexibility on mental health. A plausible explanation may be related to the various processes of psychological flexibility (e.g., perspective-taking, acceptance and value clarifications) can increase one’s prosocial behaviour. For example, those who are more psychologically flexible may acquire better perspective-taking skills, that is better attention capacities to observe other peoples’ needs during the COVID-19 pandemic, understanding the suffering of others and responding to them by helping [15, 32]. Within a helping situation, the non-judgmental acceptance attitude of a psychologically flexible person may allow a temporarily disengagement of his/her own emotions and focus on those in need of help [54]. Furthermore, engaging in values-driven behaviours is another dimension of psychological flexibility in which the meaning and underlying purpose of carrying out such behaviours often connect with prosocial-underlying values, such as kindness, caring and empathy while putting others’ interests first with some personal cost [32]. On the other hand, it is of interesting to see that higher illness perceptions toward COVID-19 was associated with better mental health through the pathway of prosociality, highlighting a plausible phenomenon that people are generally more inclined to help and support others when possessing stronger perceptions of risk related to COVID-19, in which they themselves may gain benefit in the first place with feelings of connectedness. It has been suggested that people who perceived others as having similar shared values in times of crisis are more likely to elicit a sense of common purpose in working towards the collective goal [32, 55]. For instance, seeing others in sharing resources to vulnerable population groups (e.g. elderly) during the COVID-19 pandemic may lead individuals to perceive that others as sharing the same value of self-transcendence, and hence they are more willing to self-sacrifice for the greater societal good, leading to increased feelings of connectedness and better emotional well-being [32]. As a whole, the relationship between psychological flexibility and prosociality, and their plausible benefits to one’s mental health as found in the present study, appear to be congruent with the aforementioned theoretical expectations. However, we suggest this notion can be further examined and confirmed in future research during the process of COVID-19 pandemic, and/or other disaster-related contexts (e.g., outbreaks of novel infectious diseases).

The study findings support the notion that psychological flexibility and prosociality are promising targets in an intervention for helping the public in navigating the mental health challenges regarding the pandemic. As the psychological flexibility model underpins one of the most promising approaches to cognitive behavioural therapy - Acceptance and Commitment Therapy (ACT) [44, 56], it is suggested that ACT can be used as the cornerstone of developing public mental health interventions to combat the impacts of COVID-19, which continue to unfold. Indeed, a growing body of evidence from systematic reviews have indicated the positive effects of ACT for depression [56], anxiety [56] and subjective well-being [57] through fostering psychological flexibility among clinical and non-clinical populations with small-to-medium effect sizes (Cohen’s *d* = 0.24–0.64) [56]. On the other hand, the significant association between psychological flexibility and prosociality as shown in our model further indicates that prosociality is potentially malleable through ACT leading to better mental health outcomes. In fact, interventions that stimulate prosocial behaviours have been recently reviewed by Mariola and colleagues (2020), showing that psychological approaches which focus on emotional regulation, cultivation of empathy, perspective-taking, gratitude, and compassion increase the altruistically motivated prosocial behaviours of an individual [58]. As Mariola et al.’s work did not find the use of ACT targeting at prosociality, implying the need for future ACT studies to examine whether addressing this malleable factor can nurture people with helping attitude and behaviour as an alternative way of coping. It is likely that the practice of physical distancing in containment of COVID-19 spread is longstanding. If a prosocial-oriented ACT intervention is proposed to fight the COVID-19 pandemic, alternative modes of delivery to face-to-face sessions, such as smartphone applications, video-conferencing or even self-help ACT, are recommended [21, 59]. Apart from adopting ACT interventions, a recent study has found that even a very brief psychological flexibility training, for example using one experiential metaphorical exercise that targets at practising present-moment awareness, acceptance and values clarification, has been found to increase prosocial behaviour [60].

### Limitations

Our findings need to be tempered by considering a few limitations. Although we relied on logical and theoretical basis for interpreting illness perceptions toward COVID-19 as a predictor, coping, psychological flexibility and prosociality as potential mediators and public mental health as an outcome of interest [14, 20, 34], our ability to draw robust conclusions regarding the directionality of the aforementioned constructs was restricted by the cross-sectional nature of the data. Further studies should examine our model by using longitudinal data for clarifying the relationships. Nevertheless, the significant mediating effects of psychological flexibility and prosociality acting above and beyond other coping factors may support their potential of mitigating the negative impacts of COVID-19 or other emerging infectious diseases on mental health. In this study, mental health has been assessed with the Mental Health Continuum-Short Form (MHC-SF). However, it is bear noting that mental health/well-being and mental illness/symptoms do not stand at opposite ends of the health spectrum [61], implying the need for further studies to examine whether our proposed model remains in good fit if other aspects of mental health, such as symptoms of depression and anxiety, are assessed. As indicated in one of the related reports [36], the demographic characteristics of the sample (i.e., the majority were female and attained educational level at least in college or above) and the relatively small sample size might have limited the representativeness of the study findings to Hong Kong population. Different from other cities that implement a complete/regional lock-down or massive screening, Hong Kong has been adopting a “suppress and lift” anti-pandemic strategy, in which the COVID-19 precautionary measures such as border control, physical distancing, contact tracing, and COVID-19 screening for high-risk groups are adjusted according to the incidence of infection [62]. More importantly, the strong civic unity and rapid community responses learnt from the SARS outbreak in 2003 may have contributed to the low incidence and mortality of COVID-19 in Hong Kong. In view of the large differences in terms of the COVID-19 spreading and its containment polices between Hong Kong and other regions/cities, our proposed model deserves to be further examined in other countries under the prevailing COVID-19 crisis.

## Conclusions

The current study underscores what psychological processes and coping factors play the key roles in protecting public mental health in the COVID-19 crisis. Referring to the context in Hong Kong, we identified that psychological flexibility and prosociality are the focal treatment targets, which constitute an important step toward developing mechanistically informed interventions for buffering the effects of COVID-19 and other disaster-related global stressors. The findings of our SEM imply that fostering psychological flexibility may encourage more prosocial behaviours in the communities, such as volunteering, helping others and providing caregiving beyond the family level support, for enhancing well-being amid the pandemic.

## Data Availability

The datasets used and/or analysed during the current study are available from the corresponding author on reasonable request.

## Abbreviations

ACT: Acceptance and Commitment Therapy
Brief COPE: Brief Coping Orientation to Problems Experienced
CFA: Confirmatory factor analyses
CFI: Comparative Fit Index
CI: Confidence interval
COVID-19: Coronavirus disease 2019
EPC: Expected parameter change
IPQ: Illness Perception Questionnaire
MHC-SF: Mental Health Continuum Short Form
MI: Modification index
RMSEA: Root mean square error approximation
SARS: Severe acute respiratory syndrome
SEM: Structural equation model
SRMR: Standardised root means square residual
TLI: Tucker-Lewis Index
